# Supplementary material for: Identification of photoperiod-induced specific miRNAs in the adrenal glands of Sunite sheep (Ovis aries)
Source: Front Vet Sci. 2022 Jul 22;9:888207. doi: 10.3389/fvets.2022.888207 (PMC9354845; doi:10.3389/fvets.2022.888207)
Supplement: Supplementary file 1 [file Data_Sheet_1.ZIP › Supplementary file/miRDeep2 file.zip!/miRDeep2 file/Know_Structure/LP42A3/oar-mir-200b.pdf]

miRBase precursor : oar-mir-200b
Total read count : 46
oar-miR-200b read count : 45
remaining reads : 1

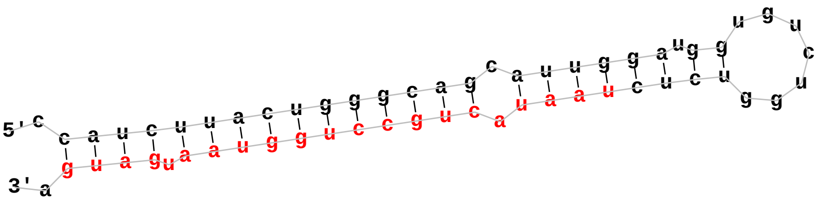

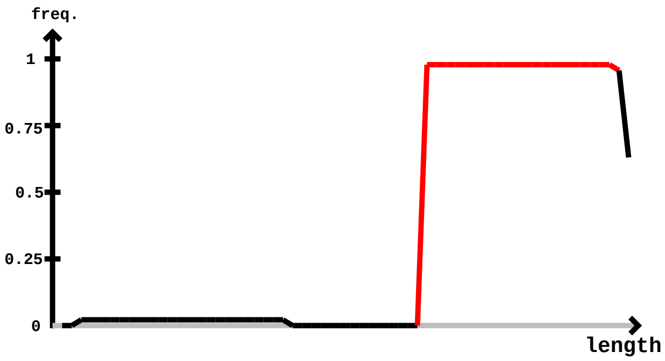

oar-miR-200b

```
5'- ccaucuuacugggcagcauuggauggugucuggucucuaauacugccugguaaugaugaa   -3'    exp
    .(((((((((((((((.((((((.((.......)))))))).))))))))))).)))).     reads      mm         sample
    .caucuuacugggcagcauugga....................................     1          0          seq
    .....................................uaauacugccugguaaugau..     1          0          seq
    .....................................uaauacugccugguaaugaug.     15         0          seq
    .....................................uaauacugccugguaaugauga     29         0          seq
```
